# Disseminated Peritoneal Leiomyomatosis: Two Rare Cases With Diagnostic Mimics and a Novel Central Nervous System Disease Association

**DOI:** 10.7759/cureus.79158

**Published:** 2025-02-17

**Authors:** Pranav S Renavikar, Thomas J Auen, Subodh M Lele, David G Wagner

**Affiliations:** 1 Pathology, Microbiology, and Immunology, University of Nebraska Medical Center, Omaha, USA

**Keywords:** hormones, leiomyomatosis, medulloblastoma, peritoneal, unique association

## Abstract

Disseminated peritoneal leiomyomatosis (DPL) is a rare benign smooth muscle tumor that proliferates along the peritoneal surface and is mostly reported in young women. Our cases highlight the wide clinical and radiologic mimics of DPL, including primary peritoneal (mesothelial) entities and malignant processes like metastasis or pseudomyxoma peritonei. Both cases shared common findings of premenopausal age (hormonal influence) and a history of prior abdominal surgery. One case was clinically thought to be benign multicystic mesothelioma, while the other had a history of medulloblastoma as a child, followed by recurrent meningiomas. The presentation of multiple previous tumors in the latter case is an unusual association with DPL that has not been previously described. Here, we discuss the existing literature on the etiology and differential diagnosis of DPL, report our histopathologic findings, and highlight novel central nervous system (CNS) disease associations with DPL.

## Introduction

Disseminated peritoneal leiomyomatosis (DPL) is a rare benign smooth muscle tumor that proliferates along the peritoneal surface and is mostly reported in young women. Due to the extensive nature of the tumor, the initial diagnostic impression can often be confused with lesions like mesothelioma, gastrointestinal stromal tumor, pseudomyxoma peritonei, and malignant metastases [1], leading to intraoperative surprises and treatment reconsiderations. Prolonged hormonal exposure leading to peritoneal mesenchymal metaplasia or iatrogenic implantation of smooth muscle cells are the widely supported theories for the development of DPL [2,3]. We describe two unique cases that support the multifactorial theory of DPL development and highlight the diagnostic dilemma. One of our patients had a history of multiple pregnancies (approximately five and two years prior to presentation) and myomectomy (approximately six years prior to presentation); however, the clinico-radiological impression was of benign multicystic mesothelioma. The second patient interestingly had a history of medulloblastoma as a child, followed by recurrent meningiomas, and later presented with DPL. To our knowledge, such an incidental but unique association between central nervous system (CNS) tumors and DPL has not been published before. In this report, we analyze and review the literature on differential diagnoses and histopathology and shed light on novel disease associations of DPL.

## Case presentation

Case 1

Clinical Presentation

**Figure 1:**
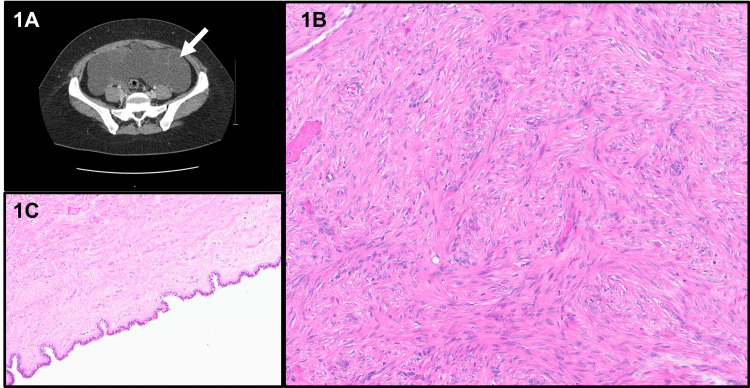
Radiology and histology findings of case 1 (1A) CT imaging revealed large, multiple multilocular cystic masses in the abdominopelvic and perihepatic areas (arrow). (1B) Representative histology showed fascicles of bland spindle cells without atypia, necrosis, or mitoses. (1C) Associated cystic endosalpingiosis was observed. These H&E stained sections were digitally scanned at 20x magnification (Aperio GT450, Leica Biosystems). CT: computed tomography; H&E: hematoxylin and eosin

A young female (late 20s; gravida 2, para 1 (G2P1)) presented to her local gynecologist for an annual exam for bloating and pelvic pain. No change in appetite or bowel habits was noted. The patient had a history of type 2 diabetes, migraine, and irregular menses. Prior surgical history included myomectomy for a uterine fibroid (via exploratory laparotomy, low-midline approach), dilatation, and curettage. Imaging studies with computed tomography (CT) and magnetic resonance imaging (MRI) were performed and revealed multiple multilocular cystic abdominopelvic and perihepatic masses (Figure 1A). Normal ovaries were identified, and the cysts did not originate from them. The appendix was not readily visualized. The differential diagnoses included a primary peritoneal process (benign multicystic peritoneal mesothelioma), appendiceal pseudomyxoma peritonei, or less likely, ovarian/gynecologic etiology. With reasonable suspicion of benign multicystic peritoneal mesothelioma (young female with prior abdominal surgery), the optimal treatment involved complete excision of the cysts, appendectomy, omentectomy, and hyperthermic intraperitoneal chemotherapy (HIPEC) with carboplatin. 

Pathology

Intraoperatively, the omentum, diaphragm, perihepatic, and periadnexal areas showed variably sized cysts, while the bowel mesentery showed fibrotic areas. The anterior surface of the uterus had nodularity. Frozen section diagnosis on one of the perihepatic lesions was rendered as low-grade mesenchymal cells. The ovaries appeared normal and were left in situ.

**Figure 2:**
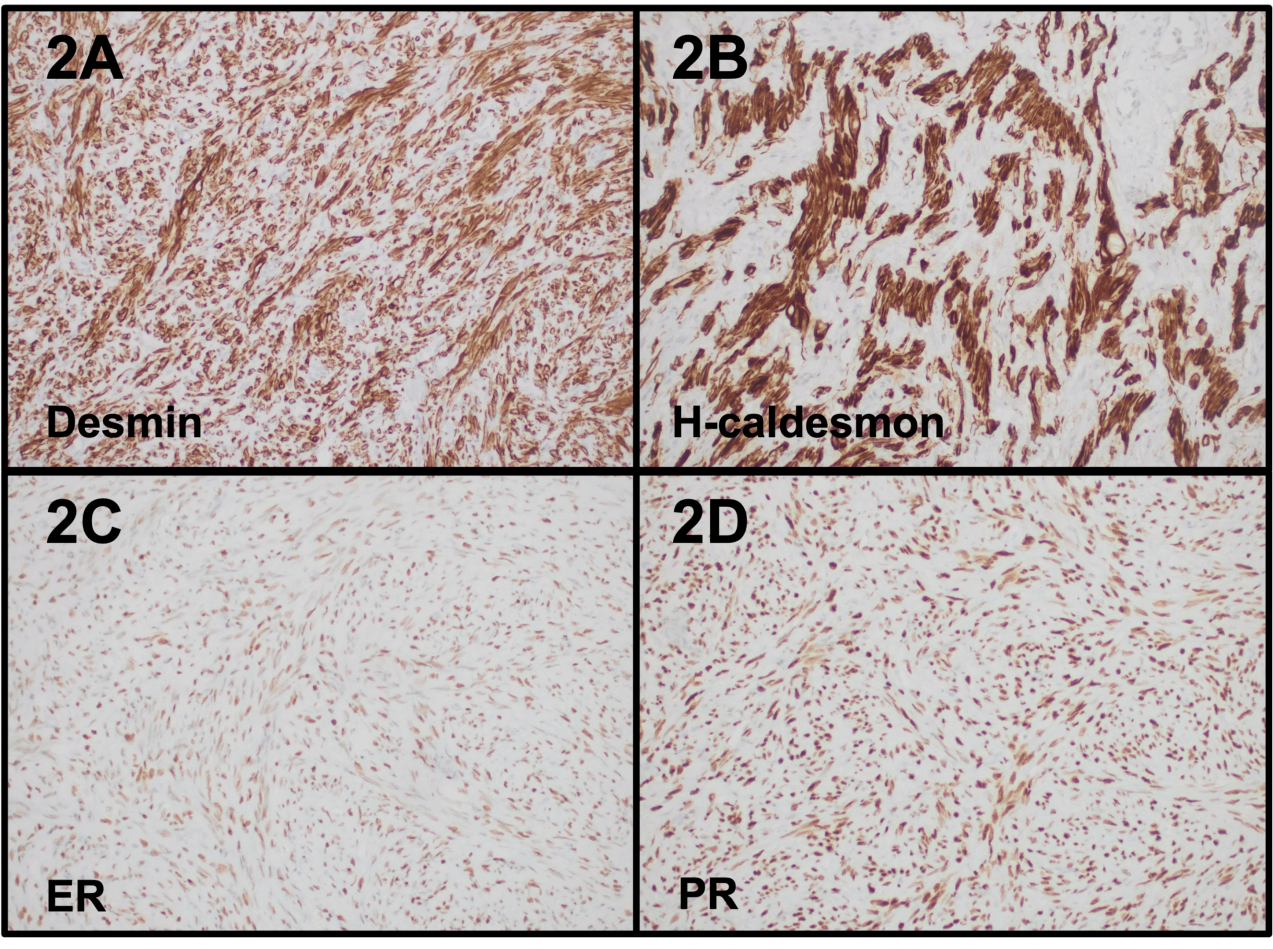
Immunohistochemistry findings of case 1 The immunohistochemical studies showed the lesional cells to be positive for desmin (2A), h-caldesmon (2B), ER (100%) (2C), and PR (100%) (2D). These sections were digitally scanned at 20x magnification (Aperio GT450, Leica Biosystems). ER: estrogen receptor; PR: progesterone receptor

Histopathologic examination revealed fascicles of bland spindle cells with no atypia, necrosis, or mitoses in all the resected pelvic cysts, mesentery, and periappendiceal soft tissue (Figure 1B). Most of the cystic appearance of the lesions was due to associated endosalpingiosis (Figure 1C). The appendix was negative for malignancy. The lesional smooth muscle cells were positive for desmin, smooth muscle actin (SMA), h-caldesmon, CD10, estrogen receptor (ER) (100%), and progesterone receptor (PR) (100%), while they were negative for PAX8, WT1, calretinin, D2-40, and AE1-AE3. Pertinent immunohistochemistry is shown in Figure 2. Thus, the overall diagnosis was disseminated peritoneal leiomyomatosis.

Follow-Up

The patient made a full recovery after the debulking procedure and was followed up with regular imaging studies at an outside institute.

Case 2

Clinical Presentation

**Figure 3:**
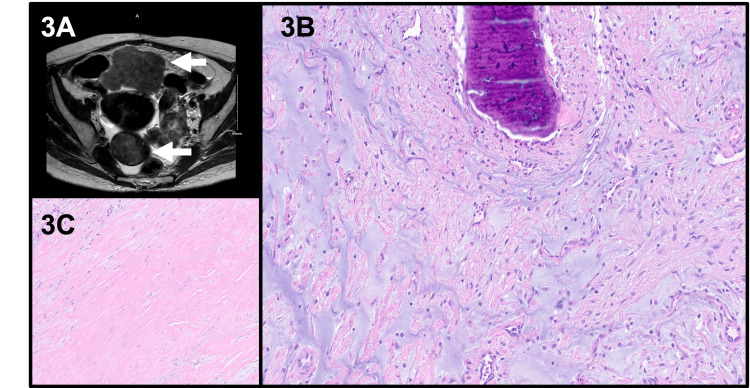
Radiology and histology findings of case 2 (3A) MRI imaging revealed multiple abdominopelvic and mesenteric solid enhancing lesions (arrows). (3B, 3C) Representative histology showed bland spindle cells with no atypia, necrosis, or mitoses. Heterogenous areas of fibromyxoid change, metaplastic bone formation, and hyalinization were observed. These H&E stained sections were digitally scanned at 20x magnification (Aperio GT450, Leica Biosystems). MRI: magnetic resonance imaging; H&E: hematoxylin and eosin

A young female (late 30s) presented with a four-month history of nausea, emesis, and unintentional weight loss. Her past medical history included medulloblastoma as a child, which was resected and treated with chemotherapy and radiation therapy. She was followed up by neurosurgery for radiation-induced recurrent meningiomas. A prior surgical history of right salpingo-oophorectomy and left ovarian cystectomy was noted. Imaging with CT and MRI scans showed multiple abdominopelvic and mesenteric solid enhancing lesions (4.5-7.0 cm) leading to pelvic ascites and gastric outlet obstruction (Figure 3A). The uterus demonstrated a large 14-week size with a fundic fibroid; however, the mesenteric lesions were not contiguous with the uterus. Esophagogastroscopy and colonoscopy were unremarkable. The differential diagnoses included disseminated peritoneal leiomyomatosis, gastrointestinal stromal tumor, or metastatic malignancy. Interventional radiology-guided biopsy was performed on the pelvic and abdominal masses with results consistent with leiomyoma.

The patient underwent pre-surgical hormonal therapy with leuprolide to shrink the tumor size, followed by surgical treatment with total abdominal hysterectomy, left salpingo-oophorectomy, resection of abdominopelvic masses, small bowel resection, and lysis of adhesions. The intraoperative findings confirmed fibroids in the uterus, bilateral adnexae, posterior pelvis, and small bowel mesentery.

Pathology

**Figure 4:**
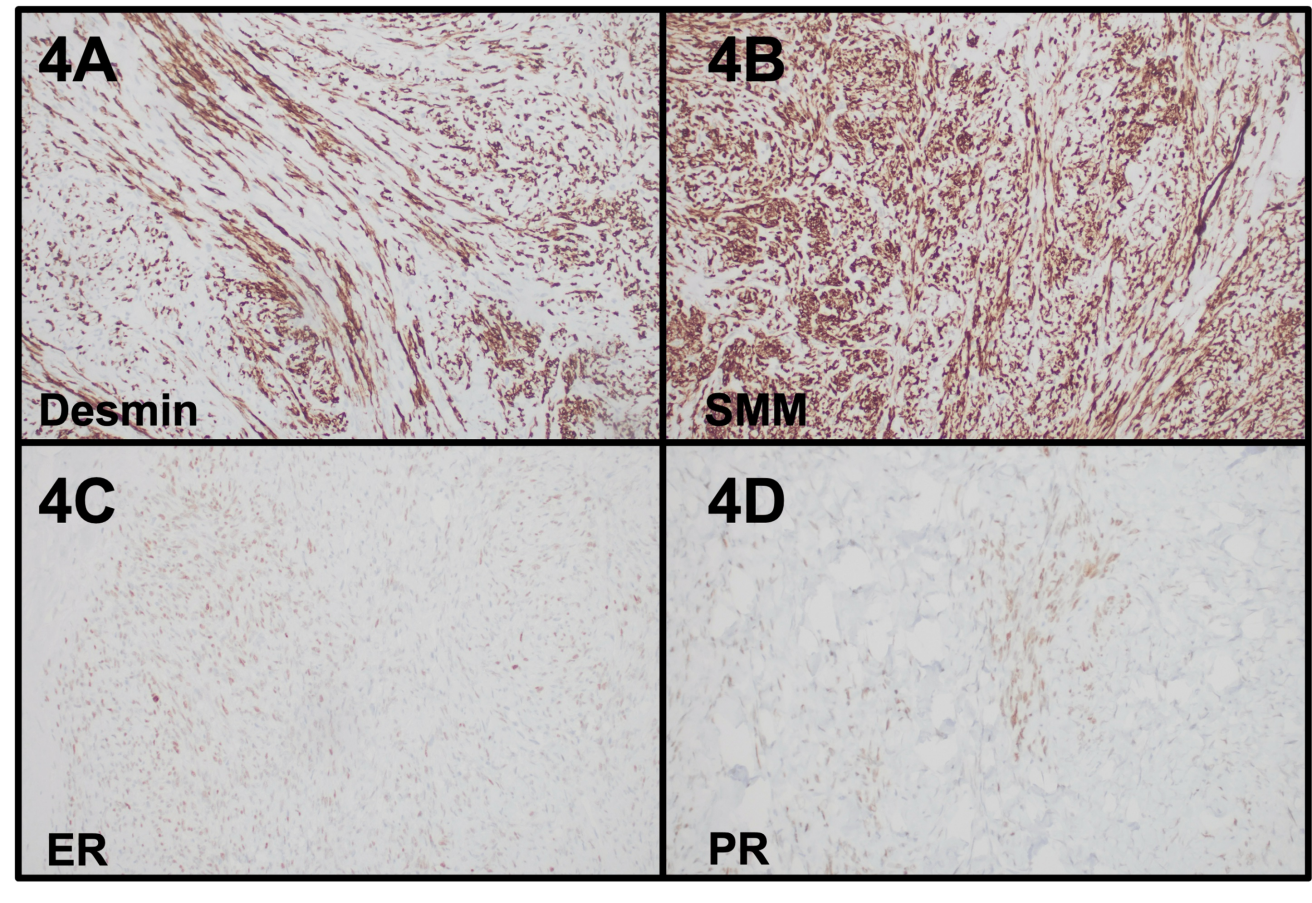
Immunohistochemistry findings of case 2 The immunohistochemical studies showed the lesional cells to be positive for desmin (4A), SMM (4B), ER (35%) (4C), and PR (15%) (4D). These sections were digitally scanned at 20x magnification (Aperio GT450, Leica Biosystems). SMM: smooth muscle myosin; ER: estrogen receptor; PR: progesterone receptor

On histopathology, all the resected specimens were consistent with a bland spindle cell proliferation. Some masses showed areas of hyalinization, degenerative and fibromyxoid change, and metaplastic bone formation (Figures 3B-3C). No cytologic atypia, necrosis, or increased mitoses were seen. Immunohistochemistry showed the spindle cells expressing SMA, desmin, and smooth muscle myosin (SMM), while they were negative for CD117, DOG1, S100, and CD34. WT1, ER (35%), and PR (15%) were expressed on the uterine and adnexal masses but were negative on the mesenteric masses. Pertinent immunohistochemistry is shown in Figure 4. Thus, the diagnosis of disseminated peritoneal leiomyomatosis was confirmed.

Follow-Up

The patient was followed up with CT scans at three-month intervals, with the latest scan showing stable non-symptomatic residual masses in the deep umbilical, mid, and right upper quadrant mesentery (2.1-4.0 cm). Resection was not advised to preserve bowel function. For residual disease, the patient was supplemented with an aromatase inhibitor (letrozole).

## Discussion

Since its first description by Taubert et al. [4], DPL has been extensively published worldwide in case reports. The rarity of this entity highlights the elevated risk of misdiagnosis and its underestimated incidence, both due to a lack of typical clinical symptoms [5]. While DPL is typically a benign diagnosis, a small subset of cases has demonstrated malignant transformation with widespread impact on multiple organ systems [6]. This suggests a need to improve the ways in which DPL is diagnosed and understood at the pathogenic level. 

Multiple etiologic theories have been proposed about DPL [7]. The first theory involves an iatrogenic implantation of smooth muscle cells secondary to surgical intervention. This has been cited with increasing frequency due to the introduction of laparoscopic power morcellation of symptomatic uterine leiomyomas compared to conventional myomectomy or hysterectomy procedures [2,3]. Laparoscopic morcellation of leiomyomas without containment techniques has been shown to be associated with an increased risk of DPL, mainly in women of reproductive age. As such, contained morcellation techniques are recommended to prevent this complication [8]. The second theory highlights hormonal effects from exogenous or endogenous female gonadal steroid sources, which stimulate smooth muscle cell differentiation or metaplasia of the peritoneal mesenchymal stem cells [9]. This is evidenced in the increased rate of identification in reproductive-age patients, those taking contraceptive medication, or those experiencing hormone fluctuations during pregnancy. Initial contradiction to this theory was stated by Parmley et al. (1975), who rather suggested that DPL is a fibrosing deciduosis or a benign reparative process in which fibroblasts replace sub-peritoneal decidua, likely developed from proliferative metaplasia of the sub-peritoneal stroma during or around pregnancy [10]. La Greca et al. described a rare phenomenon of endomyometriosis (endometrial glands, stroma, and smooth muscle) in association with DPL and highlighted that both iatrogenic implantation and Müllerian-type metaplasia of the sub-peritoneal mesenchyme influence the development of DPL [11]. They suggested that these lesions may represent a spectrum of Müllerian-type metaplasia, from which the smooth muscle component of DPL may potentially be derived. This is supported by the coexistence of DPL with Müllerian lesions like endometriosis or endosalpingiosis in our case as well. The third theory suggests genetic tendencies toward leiomyoma development implicated through molecular alterations in MED12 and HMGA2 genes [12].

Our two described cases highlight the unique presentation of DPL within one hospital setting. The first patient provides an example of multiple factors likely to have influenced the development of her diagnosis of DPL. In the years leading up to her diagnosis, she had a surgical history of uterine myomectomy, dilatation, and curettage. The combination of surgical intervention for leiomyomas and hormonal stimulation from multiple pregnancies (G2P1) are considerable factors in the etiology of her DPL. Interestingly, the initial diagnostic impression was a benign multicystic peritoneal mesothelioma (peritoneal inclusion cysts) based on clinical symptoms and imaging findings. This plausible mimic has been previously described and can confound the workup of DPL. The initial radiographic description of multiple non-enhancing cystic lesions differed from the referenced imaging findings of DPL, which are firm, solid peritoneal lesions that demonstrate hypodensity with uniform enhancement. However, degenerative changes in these lesions can show heterogeneous enhancement [13-15], and our case highlights that cystic change can be induced due to associated endosalpingiosis. The pathological interpretation that followed provided the definite diagnosis of DPL. The differential diagnoses included peritoneal carcinomatosis (pseudomyxoma peritonei), which previous studies have described as a close mimic, clinically and radiologically. The clinical presentation of multiple pelvic/peritoneal masses raises the suspicion of peritoneal carcinomatosis [16], which needs to be ruled out by appropriate pre- and intra-operative examination of the ovaries and appendix, as was done in our case. Another lesion to consider in the differential diagnoses is benign metastasizing leiomyoma (BML). Clinically, it presents as multiple pulmonary nodules, with occasional distant metastases to the skin, soft tissue, mediastinum, etc. Peritoneal involvement is typically not a feature of this entity [17].

The second patient was also of premenopausal age but had no history of contraceptive therapy or pregnancies. She did have prior surgery, specifically a right salpingo-oopherectomy and a left ovarian cystectomy more than a decade ago. However, the surgical intervention did not involve the theorized leiomyoma morcellation procedures presumed to disseminate smooth muscle cell population throughout the peritoneum, leading to DPL development. Notably, her unique clinical history involving medulloblastoma status post-resection and chemoradiation, followed by recurrent radiation-induced meningiomas, is an unusual incidental observation at this time and will require more cases with a molecular assessment to establish any genetic link. Medulloblastoma can be one of the manifestations of Gorlin syndrome (PTCH1 alterations), and previous reports have described these patients as developing leiomyomas in multiple visceral organs [18]. Other examples of syndromes with leiomyomas as one of the manifestations include hereditary leiomyomatosis and renal cell carcinoma, which is characterized by germline alterations in the FH gene, leading to cutaneous leiomyomas, uterine leiomyomas, and papillary renal cell carcinoma [19], as well as Alport syndrome (COL4A5 and COL4A6 alterations), which is characterized by nephropathy, deafness, ocular anomalies, and diffuse leiomyomatosis that can involve the esophagus, tracheobronchial region, and uterus [20].

Overall, these two cases of DPL offer a unique glance at this rare entity and demonstrate that it creates diagnostic confusion with other conditions such as benign multicystic peritoneal mesothelioma, gastrointestinal stromal tumor, and pseudomyxoma peritonei due to extensive peritoneal involvement. Importantly, we describe a novel combination of peritoneal leiomyomatosis and CNS tumors (medulloblastoma and meningioma) in the same patient at different time points. Histopathology on biopsy or resected tissue remains the gold standard for diagnosis of DPL. Immunohistochemistry for estrogen and progesterone receptors showed variable staining - ranging from negative to weak and positive - across the two cases, validating differing pathways of DPL development. Therapy guidelines for this entity are limited. Both of our cases were treated with surgical excision; the first patient received intraperitoneal chemotherapy, while the second patient received pre and post-operative hormonal therapy, both with favorable responses. Molecular assessment of the tumor was not pursued in the current study due to clinical stability post-treatment. In this report, we provide additional knowledge about this entity to aid in accurate diagnosis and treatment.

## Conclusions

In this report, we have presented two cases of DPL and discussed diagnostic mimics, including benign multicystic peritoneal mesothelioma, gastrointestinal stromal tumor, and pseudomyxoma peritonei, due to extensive peritoneal involvement. Additionally, we have highlighted a novel association between peritoneal leiomyomatosis and CNS tumors (medulloblastoma and meningioma) in one of our patients.
